# Supplementary material for: Ultrasonic super-oscillation wave-packets with an acoustic meta-lens
Source: Nat Commun. 2019 Jul 30;10:3411. doi: 10.1038/s41467-019-11430-3 (PMC6667482; doi:10.1038/s41467-019-11430-3)
Supplement: Supplementary file 1 — Supplementary Information [file 41467_2019_11430_MOESM1_ESM.pdf]

Supplementary Information of "Ultrasonic Super-oscillation Wave-packets with an Acoustic Meta-lens"

Ya-Xi Shen[1*], Yu-Gui Peng[1,2*], Feiyan Cai[3†], Kun Huang[4], De-Gang Zhao[1], Cheng-Wei Qiu[2†], Hairong Zheng[3†], and Xue-Feng Zhu[1†]

[1]*School of Physics and Innovation Institute, Huazhong University of Science and Technology, Wuhan, 430074, China*

[2]*Department of Electrical and Computer Engineering, National University of Singapore, Singapore 117583, Republic of Singapore*

[3]*Paul C. Lauterbur Research Center for Biomedical Imaging, Institute of Biomedical and Health Engineering, Shenzhen Institutes of Advanced Technology, Chinese Academy of Science, Shenzhen 518055, China*

[4]*Department of Optics and Optical Engineering, University of Science and Technology of China, Hefei, Anhui 230026, China*

[*]Y. X. S. and Y. G. P. are equally contributed to this work.

[†]To whom correspondence should be addressed. Emails: fy.cai@siat.ac.cn (F. Y. C.); eleqc@nus.edu.sg (C. W. Q); hr.zheng@siat.ac.cn (H. R. Z.); xfzhu@hust.edu.cn (X. F. Z.)

Supplementary Figures 1 to 7 --2

Supplementary Table 1 | The structural parameters of the meta-lens ----------------6

Supplementary Note 1 | Convolution and deconvolution -----------------------------7

Supplementary Note 2 | Local wavenumber near the super-oscillation region -----8

Supplementary Note 3 | The governing equations in fluid ---------------------------8

Supplementary Note 4 | The calculation of acoustic radiation force ----------------11

Supplementary Videos 1 and 2 ---15

Supplementary Figures

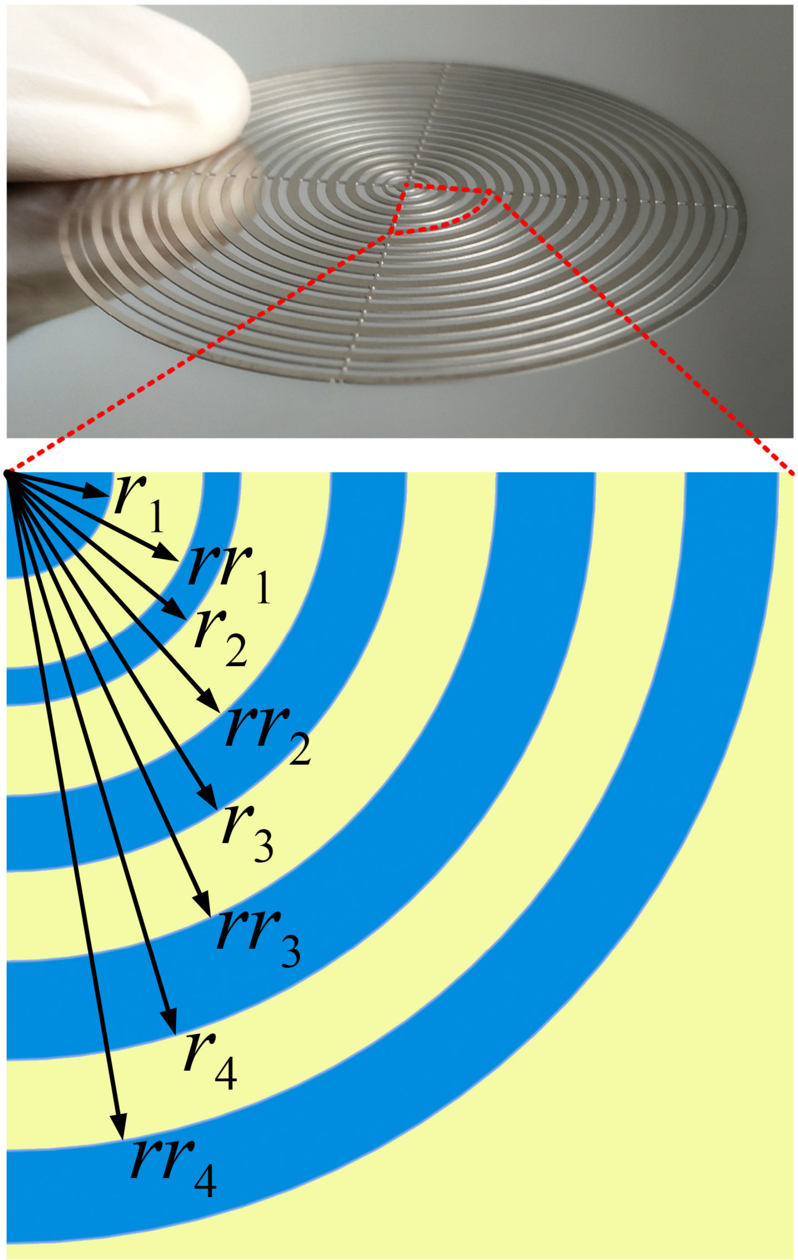

Supplementary Figure 1 | The ultrasonic meta-lens. The meta-lens is fabricated via the technique of molecular etching. The structural parameters are marked by the arrows, with $\{r_n\}_{n=1}^{N}$ and $\{rr_n\}_{n=1}^{N}$ the inner and outer radii of the circular slits (N the number of slits).

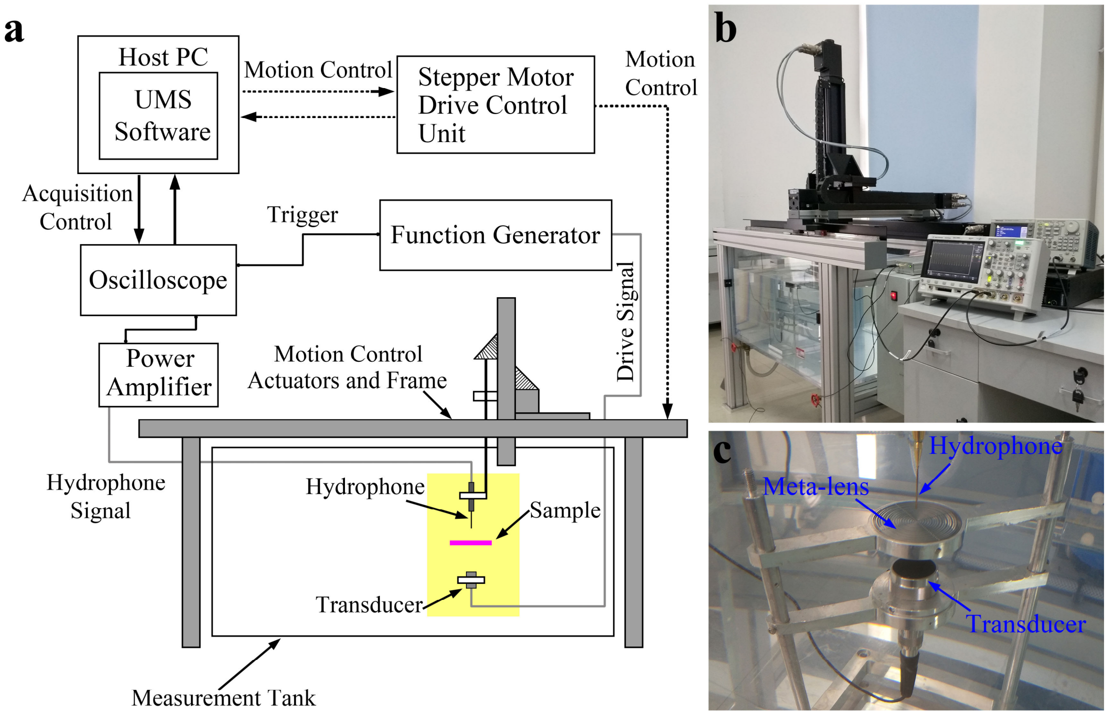

Supplementary Figure 2 | The field measurement setup. a, Schematic of the UMS 3 scanning system. **b,** Photograph of the UMS 3 scanning system. In this work, the operation frequency is 1 MHz. **c,** The zooming-in display of the field scanning region.

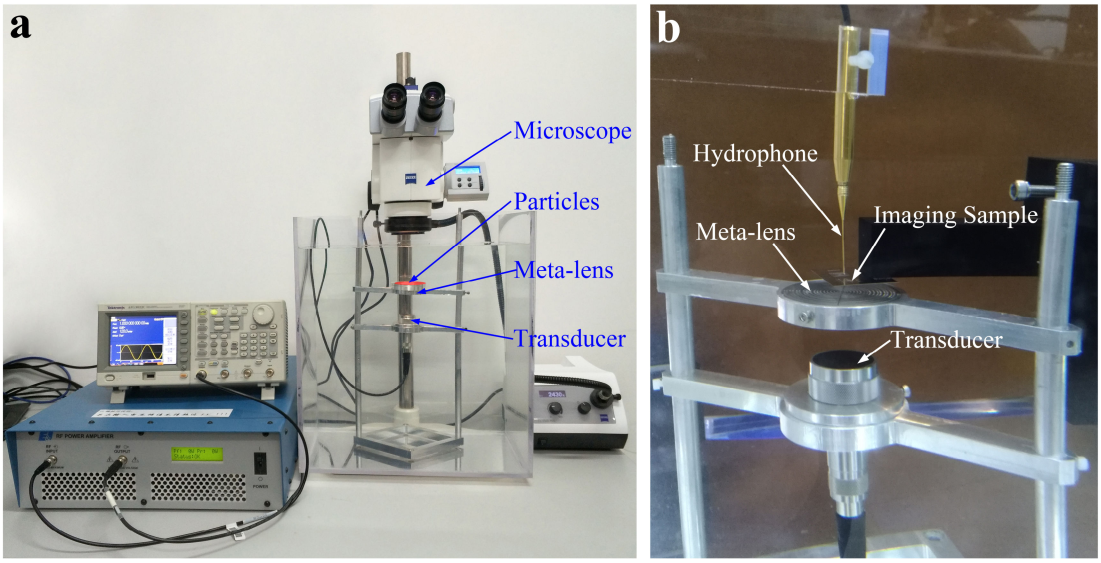

Supplementary Figure 3 | Acoustic set-ups for experiments. a, Experiment setup for acoustic tweezing. **b,** Experiment setup for super-resolution imaging.

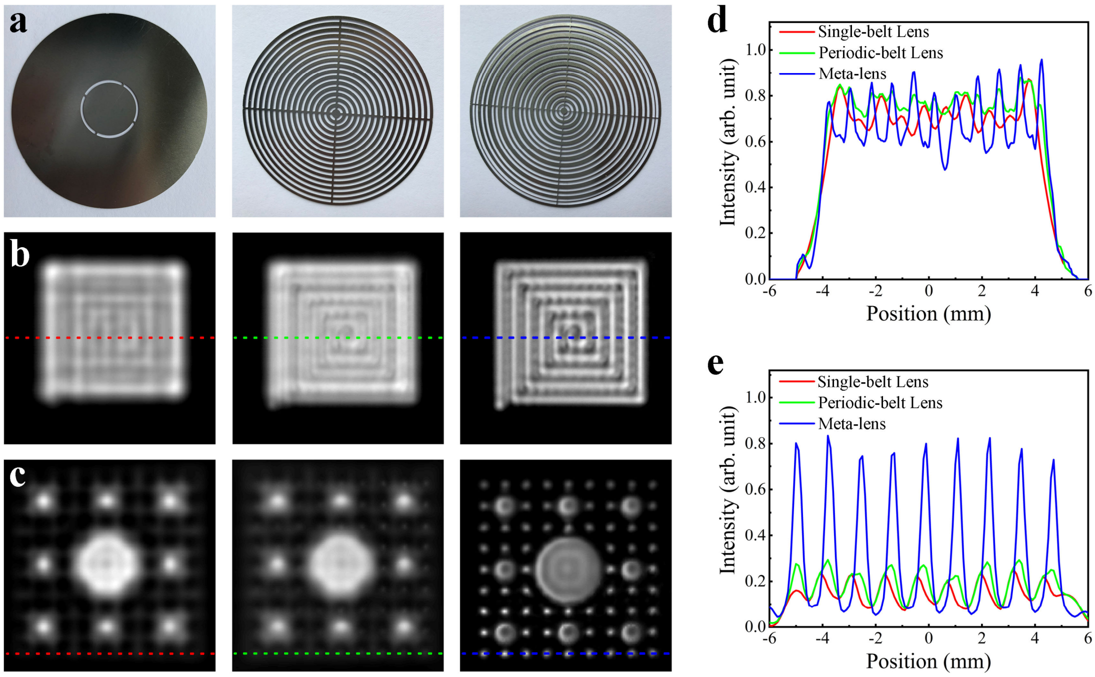

Supplementary Figure 4 | The imaging comparison of different lenses. a, The fabricated samples of a single-belt lens (left), a periodic-belt lens (middle) and a super-oscillation meta-lens (right). **b,** Ultrasound images of a coiled slit. Left: conventional imaging via a single-belt Fresnel zone plate. Middle: conventional imaging via a periodic-belt Fresnel zone plate. Right: super-resolution imaging via a meta-lens. **c,** Ultrasound images of a hole array. Left: conventional imaging via a single-belt Fresnel zone plate. Middle: conventional imaging via a periodic-belt Fresnel zone plate. Right: super-resolution imaging via a meta-lens. **d,** The intensity distributions along the dashed lines in **b**. **e,** The intensity distributions along the dashed lines in **c**. For the single-belt Fresnel zone plate, the radius $R_n = 7.5\text{mm}$ the width $\Delta r = 0.75\text{mm}$ the imaging plane locates at $z = 5.2\lambda$ with the focused sound intensity satisfying $I = C_n \,|\, J_0(kr\sin\alpha_n)|^2$. For the periodic-belt Fresnel zone plate, the period $p = 1.65\text{mm}$ the width $\Delta r = 0.75\text{mm}$ the imaging plane locates at $z = 5.2\lambda$.

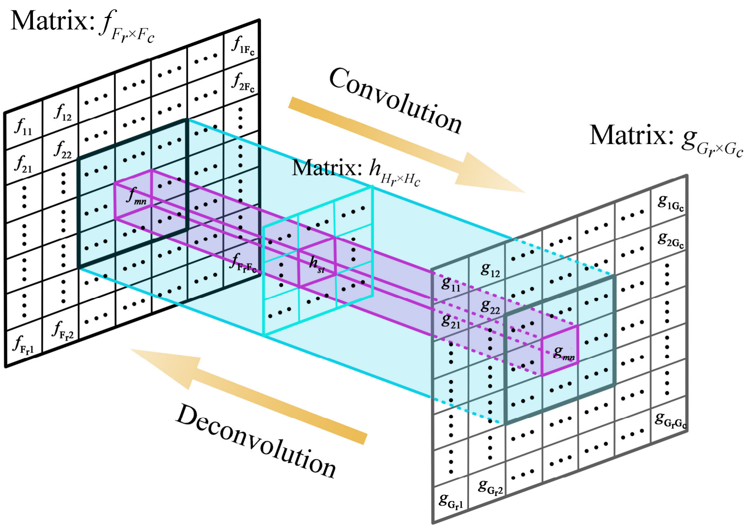

Supplementary Figure 5 | The deconvolution in pressure field post-processing. In experimental measurements, the measured field distribution is the solution function $g_{G_r \times G_c}$. The aperture function of hydrophone is the convolutional interaction function $h_{H_r \times H_c}$. The real pressure field distribution is the source function $f_{F_r \times F_c}$.

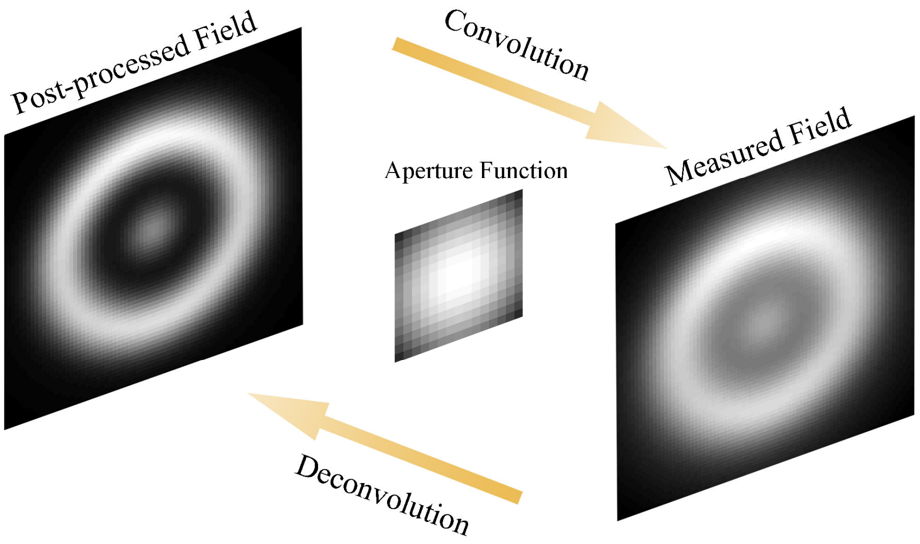

Supplementary Figure 6 | The deconvolution in deciphering the super-oscillation field. Left: the post-processed intensity field of the super-oscillation packet. Middle: the aperture function of hydrophone. Right: the measured intensity field in experiments, which is actually the convolution between the post-processed field and the aperture function.

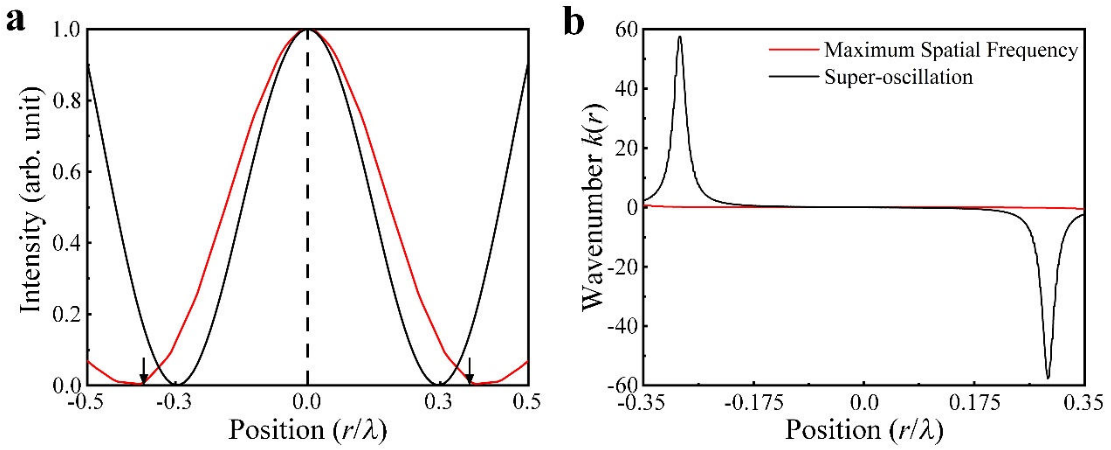

Supplementary Figure 7 | The intensity curves and local wavenumbers. a, The normalized intensity distributions of a super-oscillatory field (black line) and the maximum spatial frequency component (red line). The bottom arrows indicate the location of the diffraction limit (0.38λ). **b**, The calculated local wavenumbers of a super-oscillatory field (black line) and the maximum spatial frequency component (red line).

Supplementary Table 1 | The structural parameters of the meta-lens (Unit/mm)

$\Delta r_n = 0.75$					
R_1	1.305	R_7	10.195	R_{13}	19.645
R_2	2.405	R_8	11.545	R_{14}	20.845
R_3	3.835	R_9	12.895	R_{15}	22.645
R_4	5.465	R_{10}	14.695	R_{16}	23.995
R_5	7.045	R_{11}	16.465	R_{17}	25.525
R_6	8.495	R_{12}	17.795	R_{18}	26.535

Supplementary Notes

Supplementary Note 1 | Convolution and deconvolution

In mathematics, the convolution between the functions $f(x,y)$ and $h(x,y)$ is defined by

$$\begin{aligned} g(x,y) &= f(x,y) * h(x,y) \\ &= \iint f(\xi,\eta) h(x-\xi, y-\eta) d\xi d\eta, \end{aligned} \tag{S1}$$

where * denotes the convolution operator, $f(x,y)$ is the source function before the convolution process, $h(x,y)$ is the convolutional interaction function and $g(x,y)$ is the solution function after convolution. In experimental measurements, the functions $f(x,y)$, $h(x,y)$ and $g(x,y)$ are discretized into matrices. Then the Eq. (S1) can be expressed into

$$g(s,t) = \sum_{m=0}^{F_r-1} \sum_{n=0}^{F_c-1} f(m,n) h(s-m, t-n), \tag{S2}$$

where (m,n) and (s,t) denote the element indices of the matrices $f_{F_r \times F_c}$ and $h_{H_r \times H_c}$. F_r and F_c represent the numbers of rows and columns of the source function matrix $f_{F_r \times F_c}$. The pressure field scanning is actually a convolution process, where the measured field distribution can be regarded as the solution function $g(x,y)$. The aperture function of hydrophone is the convolutional interaction function $h(x,y)$, which is a truncated Gaussian function with the FWHM ~2 mm (size of the truncated region: ~0.6 mm). The source function $f(x,y)$ is the one that reflects the real pressure field distribution, which can be solved by the deconvolution process, as schematically described in Supplementary Figures 5 and 6.

Supplementary Note 2 | Local wavenumber near the super-oscillation region

For a single-belt Fresnel zone plate, the intensity distribution at the focal region is described by $I = C_n |J_0(kr\sin\alpha_n)|^2$, where the first minimum locates at $r = 0.38\lambda/\sin\alpha_n$. Since $\sin\alpha_n \le 1$, the diffraction limit is thus $r_D = 0.38\lambda$. Supplementary Figure 7a shows the normalized intensity distributions of the super-oscillation field (black line) and the maximum spatial frequency component (red line). The recorded focal spot with the radius $\sim 0.3\lambda$ provides us a clear evidence that a super-oscillatory field is created, based on the super-oscillation criterion that $0.3\lambda < \lambda_D = 0.38\lambda$. Moreover, from the definition of local wavenumber $k(r) = \mathrm{Im}\{\partial_r[\ln p(r)]\}$, where $p(r)$ is the band-limited function, we calculated the local wavenumbers of the super-oscillation band-limited function (black line) and the maximum spatial frequency component (red line) near the super-oscillation region ($r = [-0.35\lambda, 0.35\lambda]$), as shown in Supplementary Figure 7b. The results show that the local wavenumber of super-oscillation is much larger than the one of maximum spatial frequency component at the zero-intensity position.

Supplementary Note 3 | The governing equations in fluid

In this session, we first consider the mass conservation of fluid, namely, the continuity equation. Considering a thermo-viscous compressible fluid, for which the density ρ varies as a function of space (*r*) and time (*t*) in a fixed and arbitrarily shaped fluid region Ω. The total mass (*M*) inside volume Ω can be calculated by a volume integral over the density ρ

$$M(\Omega,\ t) = \int_{\Omega} \rho(r,\ t) d\tau \ . \tag{S3}$$

Since the mass can neither appear nor disappear spontaneously in non-relativistic mechanics, $M(\Omega, t)$ only varies due to a mass flux through the surface S of the volume Ω. So we have

$$\partial_t M(\Omega,\ t) = \partial_t \int_{\Omega} \rho(r,\ t) d\tau = -\int_S (\rho \mathbf{v}) \cdot \mathbf{n} ds \ . \tag{S4}$$

Here we define $\mathbf{n}$ as the unit normal vector pointing away from the surface S. Then, we can obtain the continuity equation by applying the Gauss's theorem

$$\partial_t \rho = -\nabla \cdot (\rho \mathbf{v}) \ . \tag{S5}$$

The second governing equation is the famous Navier-Stokes equation. It is a motion equation for the Eulerian velocity field that is directly related to the conservation of momentum density $\rho \mathbf{v}$. In analogy with the mass conservation, the rate of momentum change is given by

$$\begin{aligned} \partial_t \mathbf{P} &= \partial_t \int_{\Omega} \rho(r,\ t) \mathbf{v}(r,\ t) d\tau \\ &= \int_{\Omega} [(\partial_t \rho) \mathbf{v} + \rho \partial_t \mathbf{v}] d\tau. \end{aligned} \tag{S6}$$

On the other hand, the momentum $\mathbf{P}$ can change both by the advection and by the action of forces obeying the Newton's second law. The forces can be divided into two parts which are the body force that acts on the interior of volume Ω (gravity) and the contact force that acts on the surface S of volume Ω (pressure and viscosity force). Thus, the change rate of momentum can also be written as

$$\partial_t \mathbf{P} = \int_{\Omega} \mathbf{f}^{\text{body}} d\tau - \int_S (\rho \mathbf{v}) \mathbf{v} \cdot \mathbf{n} ds - \int_S p \mathbf{n} ds - \int_S \boldsymbol{\sigma}' \cdot \mathbf{n} ds \ , \tag{S7}$$

where $\mathbf{f}^{\text{body}}$ describes an external body force density in the entire fluid body. The tensor $(\rho \mathbf{v}) \mathbf{v}$ denotes the advection of momentum $\rho \mathbf{v}$ into the volume Ω. p and $\boldsymbol{\sigma}'$

are the pressure and the viscosity stress tensor, respectively. Analyses show that the stress tensor $\boldsymbol{\sigma}'$ must be symmetric for small velocity gradients in microfluidics. The stress tensor satisfies the following equation

$$\sigma'_{ij} = \eta(\partial_j v_i + \partial_i v_j) + (\beta - 1)\eta(\partial_k v_k)\delta_{ij}, \tag{S8}$$

where the first term relates to the dynamic shear viscosity η of incompressible fluid, and the second term appears when the compressibility-induced dilatational viscosity cannot be neglected. β is the ratio of the shear viscosity to the dilatational viscosity. Then we can obtain the Navier-Stokes equation for Newtonian fluids by applying the Gauss's theorem

$$\rho\partial_t \mathbf{v} = -\nabla p - \rho(\mathbf{v}\cdot\nabla)\mathbf{v} + \eta\nabla^2\mathbf{v} + \beta\eta\nabla(\nabla\cdot\mathbf{v}). \tag{S9}$$

In the end, we deduce the energy conservation equation in the studied system. It is derived by using an approach similar to the one employed to obtain the continuity equation and the Navier-Stokes equation. We consider the change rate of energy $\partial_t E(\Omega,\ t)$ inside the volume Ω. The energy density is given by the sum of kinetic energy density $\rho v^2/2$ and the internal energy density $\rho\varepsilon$. On the other hand, the total energy E can change via the energy advection through the surface S of volume Ω, by friction forces acting on the surface as well as the thermal gradients at the surface. Therefore, we obtain

$$\begin{aligned}\partial_t E &= \int_\Omega \partial_t\left(\frac{1}{2}\rho v^2 + \rho\varepsilon\right)d\tau \\ &= -\int_S\left(\frac{1}{2}\rho v^2 + \rho\varepsilon\right)\mathbf{v}\cdot\mathbf{n}ds - \int_S p\mathbf{v}\cdot\mathbf{n}ds + \int_S(\boldsymbol{\sigma}'\cdot\mathbf{v})\cdot\mathbf{n}ds + \int_S(\kappa_{th}\nabla T)\cdot\mathbf{n}ds,\end{aligned} \tag{S10}$$

where T and κ_{th} represent the temperature and the thermal conductivity, respectively. Applying the Gauss's theorem, the Eq. (S10) becomes

$$\partial_t(\frac{1}{2}\rho v^2 + \rho\varepsilon) = \nabla\cdot[\mathbf{v}\cdot\boldsymbol{\sigma}' + \kappa_{th}\nabla T - (\frac{1}{2}\rho v^2 + \rho\varepsilon)\mathbf{v} - p\mathbf{v}]. \tag{S11}$$

Supplementary Note 4 | The calculation of acoustic radiation force

In this work, we analyze the acoustic radiation force acting on a compressible, spherical, micrometer-sized particle of radius a suspended in a thermo-viscous fluid, where the wavelength of ultrasound λ is much larger than the particle radius a, that is, $a \ll \lambda$. In this case, the micro-particle is treated as a weak point scatterer, which can be handled by the first-order scattering theory. An expression for the acoustic radiation force $\mathbf{F}^{rad}$ is obtained by considering the momentum flux density $\boldsymbol{\sigma} + (\rho\mathbf{v})\mathbf{v}$ entering the volume between the particle surface $S_0(t)$ and an arbitrary static surface S_1 enclosing the particle, where $\boldsymbol{\sigma} = \boldsymbol{\sigma}' + p\mathbf{I}$. Since the net body force on the fluid element is zero, the time-averaged change rate of momentum $\left\langle \frac{d}{dt}\mathbf{P} \right\rangle$ is expressed by

$$\begin{aligned}\left\langle \frac{d}{dt}\mathbf{P} \right\rangle &= -\left\langle \int_{S_1} [\boldsymbol{\sigma} + (\rho\mathbf{v})\mathbf{v}]\cdot\mathbf{n}ds \right\rangle + \left\langle \int_{S_0(t)} [\boldsymbol{\sigma} + (\rho\mathbf{v})\mathbf{v}]\cdot\mathbf{n}ds \right\rangle \\ &= -\left\langle \int_{S_1} [\boldsymbol{\sigma} + (\rho\mathbf{v})\mathbf{v}]\cdot\mathbf{n}ds \right\rangle - \mathbf{F}^{rad},\end{aligned} \tag{S12}$$

where $\mathbf{n}$ is the unitary normal vector pointing out of the particle surface $S_0(t)$ and out of the static enclosure surface S_1. Finally, given that the time-averaged change rate of momentum is zero for the propagating ultrasound field, we thus obtain

$$\mathbf{F}^{rad} = -\left\langle \int_{S_1} [\boldsymbol{\sigma} + (\rho\mathbf{v})\mathbf{v}]\cdot\mathbf{n}ds \right\rangle. \tag{S13}$$

By taking the second-order perturbation and using the expansions $\rho = \rho_0 + \rho_1 + \rho_2$,

$\mathbf{v} = 0 + \mathbf{v}_1 + \mathbf{v}_2$, and $\boldsymbol{\sigma} = \boldsymbol{\sigma}_0 + \boldsymbol{\sigma}_1 + \boldsymbol{\sigma}_2$, the Eq. (S13) becomes

$$\mathbf{F}^{rad} = -\int_{S_1} [\langle \boldsymbol{\sigma}_2 \rangle + \rho_0 \langle \mathbf{v}_1 \mathbf{v}_1 \rangle] \cdot \mathbf{n} ds, \tag{S14}$$

where we assumed that the time average of the first-order component of the harmonic wave is zero. In the regions sufficiently far from the boundary layers, ultrasound waves propagate like free-space mechanical waves with little damping. Therefore, the viscous and thermal effects are negligible. In this case, Eq. (S14) can be solved by placing an arbitrary static surface enclosing the particle S_1 in the far field, which is also valid in the near field by considering the thermo-viscous effect. In the far field, the governing equations in fluid are

$$\partial_t \rho = -\nabla \cdot (\rho \mathbf{v}), \tag{S15a}$$

$$\rho \partial_t \mathbf{v} = -\nabla p - \rho (\mathbf{v} \cdot \nabla) \mathbf{v}, \tag{S15b}$$

where the first-order perturbation equations are derived as follows

$$\partial_t \rho_1 = -\rho_0 \nabla \cdot \mathbf{v}_1, \tag{S16a}$$

$$\rho_0 \partial_t \mathbf{v}_1 = -c_0^2 \nabla \rho_1. \tag{S16b}$$

After taking the time derivative ∂_t of Eq. (S16a) and substituting it with Eq. (S16b), we can obtain the first-order wave equation of

$$\partial_t^2 \rho_1 = c_0^2 \nabla^2 \rho_1. \tag{S17}$$

The time-averaged second-order perturbation of the governing equation takes the form of

$$\rho_0 \nabla \cdot \langle \mathbf{v}_2 \rangle = -\nabla \cdot \langle \rho_1 \mathbf{v}_1 \rangle, \tag{S18a}$$

$$-\nabla \cdot \langle p_2 \rangle = \langle \rho_1 \partial_t \mathbf{v}_1 \rangle + \rho_0 \langle (\mathbf{v}_1 \cdot \nabla) \mathbf{v}_1 \rangle. \tag{S18b}$$

Substituting Eq. (S16b) into Eq. (S18b) with the compressibility $\kappa_0 = 1/\rho_0 c_0^2$, we

obtain

$$\langle p_2\rangle = \frac{\kappa_0}{2}\langle p_1^2\rangle - \frac{\rho_0}{2}\langle v_1^2\rangle . \tag{S19}$$

As a result, in the far field, the acoustic radiation force can be written into

$$\begin{aligned}
\mathbf{F}^{rad} &= -\int_{S_1}\left[\langle \boldsymbol{\sigma}_2' + p_2\mathbf{I}\rangle + \rho_0\langle \mathbf{v}_1\mathbf{v}_1\rangle\right]\cdot\mathbf{n}ds \\
&= -\int_{S_1}\left[\langle p_2\rangle\mathbf{n} + \left(\rho_0\langle \mathbf{v}_1\mathbf{v}_1\rangle\right)\cdot\mathbf{n}\right]ds \\
&= -\int_{S_1}\left[\left(\frac{\kappa_0}{2}\langle p_1^2\rangle - \frac{\rho_0}{2}\langle v_1^2\rangle\right)\mathbf{n} + \left(\rho_0\langle \mathbf{v}_1\mathbf{v}_1\rangle\right)\cdot\mathbf{n}\right]ds.
\end{aligned} \tag{S20}$$

On the other hand, the first-order perturbations of pressure p_1 and velocity $\mathbf{v}_1$ can be expressed in terms of the first-order perturbation of velocity potential ϕ_1 as $p_1 = -\rho_0\partial_t\phi_1$ and $\mathbf{v}_1 = \nabla\phi_1$, respectively. According to the standard scattering theory, the velocity potential of the scattered field ϕ_{sc} from a point particle can be represented by a time-retarded multipole expansion. In the far field, the monopole and dipole components dominate the scattered field, that is $\phi_{\text{sc}} = \phi_{\text{mp}} + \phi_{\text{dp}}$. In ultrasound fields, the total velocity potential ϕ_1 is the summation of the velocity potentials of incident and scattered fields (ϕ_{in} and ϕ_{sc}). Therefore, we have

$$\phi_1 = \phi_{\text{in}} + \phi_{\text{sc}} , \tag{S21}$$

with

$$\phi_{\text{sc}} = -f_1\frac{a^3}{3\rho_0}\frac{\partial_t\rho_{\text{in}}(t-r/c_0)}{r} - f_2\frac{a^3}{2}\nabla\cdot\left[\frac{\mathbf{v}_{\text{in}}(t-r/c_0)}{r}\right],\ \ r \ll \lambda , \tag{S22}$$

where a, f_1, f_2 are the radius of particles, the scattering coefficients of monopole and dipole components, respectively. By inserting Eq. (S21) into Eq. (S20), we will obtain a sum of terms for acoustic radiation force $\mathbf{F}^{rad}$, with each term proportional to $(\phi_{\text{in}} + \phi_{\text{sc}})^2$. The result indicates that there exist three parts of contributions to the

acoustic radiation force, *i.e.*, (i) ϕ_{in}^2 that contains no information about the scattering component and thus contributes zero to the force, (ii) ϕ_{sc}^2 that is proportional to the square of particle volume (a^6) and is therefore negligible, (iii) $\phi_{\text{in}}\phi_{\text{sc}}$ that is proportional to particle volume (a^3) and dominantly contributes to $\mathbf{F}^{rad}$. By keeping only the mixed term $\phi_{\text{in}}\phi_{\text{sc}}$ and using the index notation, the *i*-th component of Eq. (S20) becomes

$$\begin{aligned}
F_i^{rad} &= -\int_{S_1}\left[\left(\frac{c_0^2}{\rho_0}\langle\rho_{\text{in}}\rho_{\text{sc}}\rangle - \rho_0\langle v_k^{\text{in}} v_k^{\text{sc}}\rangle\right)\delta_{ij} + \rho_0\langle v_i^{\text{in}} v_j^{\text{sc}}\rangle + \rho_0\langle v_i^{\text{sc}} v_j^{\text{in}}\rangle\right] n_j ds \\
&= -\int_{\Omega}\left[\frac{c_0^2}{\rho_0}(\langle\rho_{\text{in}}\partial_i\rho_{\text{sc}}\rangle + \langle\rho_{\text{sc}}\partial_i\rho_{\text{in}}\rangle) + \rho_0(\langle v_i^{\text{in}}\partial_j v_j^{\text{sc}}\rangle + \rho_0\langle v_i^{\text{sc}}\partial_j v_j^{\text{in}}\rangle)\right] d\tau \\
&= -\int_{\Omega}(-\langle\rho_{\text{in}}\partial_t v_i^{\text{sc}}\rangle - \langle\rho_{\text{sc}}\partial_t v_i^{\text{in}}\rangle + \rho_0\langle v_i^{\text{in}}\partial_j v_j^{\text{sc}}\rangle - \langle v_i^{\text{sc}}\partial_t\rho_{\text{in}}\rangle) d\tau \\
&= -\int_{\Omega}(\langle v_i^{\text{in}}\partial_t\rho_{\text{sc}}\rangle + \rho_0\langle v_i^{\text{in}}\partial_j v_j^{\text{sc}}\rangle) d\tau \\
&= -\int_{\Omega}\rho_0\left\langle v_i^{\text{in}}\left(\partial_j^2 - \frac{1}{c_0^2}\partial_t^2\right)\phi_{\text{sc}}\right\rangle d\tau.
\end{aligned} \tag{S23}$$

After reformulation, we will obtain

$$\mathbf{F}^{rad} = -\int_{\Omega}\rho_0\left\langle \mathbf{v}_{in}\left(\nabla^2 - \frac{1}{c_0^2}\partial_t^2\right)\phi_{sc}\right\rangle d\tau. \tag{S24}$$

Substituting Eq. (S22) into Eq. (S24), we will obtain

$$\begin{aligned}
\mathbf{F}^{rad} &= -\int_{\Omega}\rho_0\left\langle \mathbf{v}_{\text{in}}\left\{f_1\frac{4\pi a^3}{3\rho_0}\partial_t\rho_{\text{in}}\delta(\mathbf{r}) + f_2 2\pi a^3\nabla\cdot[\mathbf{v}_{\text{in}}\delta(\mathbf{r})]\right\}\right\rangle d\tau \\
&= -\frac{4\pi a^3}{3}\langle f_1\mathbf{v}_{\text{in}}\partial_t\rho_{\text{in}}\rangle + 2\pi a^3\rho_0\langle f_2(\mathbf{v}_{\text{in}}\cdot\nabla)\mathbf{v}_{\text{in}}\rangle \\
&= \frac{4\pi a^3}{3}\langle f_1\rho_{\text{in}}\partial_t\mathbf{v}_{\text{in}}\rangle + 2\pi a^3\rho_0\langle f_2(\mathbf{v}_{\text{in}}\cdot\nabla)\mathbf{v}_{\text{in}}\rangle \\
&= -\frac{4\pi a^3}{3\rho_0 c_0^2}\langle f_1 p_{\text{in}}\nabla p_{\text{in}}\rangle + 2\pi a^3\rho_0\langle f_2(\mathbf{v}_{\text{in}}\cdot\nabla)\mathbf{v}_{\text{in}}\rangle \\
&= -\pi a^3\left[\frac{2\kappa_0}{3}\text{Re}(f_1^* p_{in}^*\nabla p_{in}) - \rho_0\,\text{Re}(f_2^*\mathrm{v}_{in}^*\cdot\nabla\mathrm{v}_{in})\right],
\end{aligned} \tag{S25}$$

where $\mathbf{v}_{\mathrm{in}} = -\frac{1}{\rho_0}\int \nabla p_{in} dt$ and p_{in} is the incident pressure field evaluated at $\mathbf{r} = 0$. From Eq. (S25), the acoustic radiation force $\mathbf{F}^{rad}$ is expressed in terms of the velocity potential of incident acoustic wave ϕ_{in} at the particle position as well as the scattering coefficients of the monopole and dipole components, namely, f_1 and f_2. Based on the previous study, the monopole scattering coefficient $f_1 = 1 - \frac{\rho_0 c_0^2}{\rho_p c_p^2}$ and dipole scattering coefficient $f_2 = \frac{2(\rho_p - \rho_0)}{2\rho_p + \rho_0}$, where c_0 and ρ_0 are the sound speed and the density of water, c_p and ρ_p denote the sound speed and the density of tiny particles.

Supplementary Video 1. A video made to show the dynamic evolution of the constructed super-oscillatory function in the time range of $t = 0 \sim 1.39\pi$ (unit: second).

Supplementary Video 2. A video made via a stereomicroscope (ZEISS V20) to real-time monitor the movement of micro-particles (the mean diameter ~100μm). The operation frequency is 1 MHz. We switched on and off the power source to reproduce the effect of ultrasound super-oscillation tweezing.
