# Supplementary material for: Ultrasonic super-oscillation wave-packets with an acoustic meta-lens
Source: Nat Commun. 2019 Jul 30;10:3411. doi: 10.1038/s41467-019-11430-3 (PMC6667482; doi:10.1038/s41467-019-11430-3)
Supplement: Supplementary file 4 — Description of Additional Supplementary Files [file 41467_2019_11430_MOESM4_ESM.docx]

**Title:** Supplementary Video 1
**Description:** Show the dynamic evolution of the constructed super-oscillatory function in the time range of $t=0\sim1.39\pi$(unit: second).

**Title:** Supplementary Video 2
**Description:** Made via a stereomicroscope (ZEISS V20) to real-time monitor the movement of micro-particles (the mean diameter $\sim100\text{μm}$). The operation frequency is 1 MHz. We switched on and off the power source to reproduce the effect of ultrasound super-oscillation tweezing.
